# The structural effect between the output module and chromophore-binding domain is a two-way street via the hairpin extension

**DOI:** 10.1007/s43630-022-00265-5

**Published:** 2022-08-19

**Authors:** Moona Kurttila, Stefan Etzl, Jessica Rumfeldt, Heikki Takala, Nadine Galler, Andreas Winkler, Janne A. Ihalainen

**Affiliations:** 1 Nanoscience Center, Department of Biological and Environmental Science, University of Jyväskylä, 40014 Jyväskylä, Finland; 2 Institute of Biochemistry, Graz University of Technology, Petersgasse 12/II, 8010 Graz, Austria

## Abstract

Signal transduction typically starts with either ligand binding or cofactor activation, eventually affecting biological activities in the cell. In red light-sensing phytochromes, isomerization of the bilin chromophore results in regulation of the activity of diverse output modules. During this process, several structural elements and chemical events influence signal propagation. In our study, we have studied the full-length bacteriophytochrome from *Deinococcus radiodurans* as well as a previously generated optogenetic tool where the native histidine kinase output module has been replaced with an adenylate cyclase. We show that the composition of the output module influences the stability of the hairpin extension. The hairpin, often referred as the PHY tongue, is one of the central structural elements for signal transduction. It extends from a distinct domain establishing close contacts with the chromophore binding site. If the coupling between these interactions is disrupted, the dynamic range of the enzymatic regulation is reduced. Our study highlights the complex conformational properties of the hairpin extension as a bidirectional link between the chromophore-binding site and the output module, as well as functional properties of diverse output modules.

**Supplementary Information:**

The online version contains supplementary material available at 10.1007/s43630-022-00265-5.

## Introduction

Protein function is influenced to a large degree by the biophysical properties of the 20 proteinogenic amino acids and their 3-dimensional arrangement. While a diversity of ligand-binding sites or active centers can be formed this way, the versatility of protein function can be even further expanded by interactions of proteins with non-protein-based organic compounds or metal ions—so called cofactors. Not only do they provide access to chemistry otherwise infeasible, but they also enable photoreceptor proteins to sense light ranging from the blue region all the way to the near-infrared end of the electromagnetic spectrum [41]. In the case of photoreceptors, cofactors not only need to absorb light of their corresponding spectral regions, but they also should allow signal integration via functional interactions with regulatory parts of the protein. To this end, characteristic hydrogen-bonding interactions are typically formed with polar amino acid side chains or water molecules in the cofactor binding site. Eventually, these interactions can be modulated during the formation of a photoactivated state and, subsequently, the local structural rearrangements can be propagated over long distances to enable allosteric regulation of different output functionalities or modulation of biological interactions [14]. These light-induced structural rearrangements are an active area of research but their description at a molecular level is frequently complicated by the dynamic nature of these processes. A comprehensive understanding requires an integrative approach that enables analysis over different time and length scales [8, 19, 23, 25, 49].

**Fig. 1:**
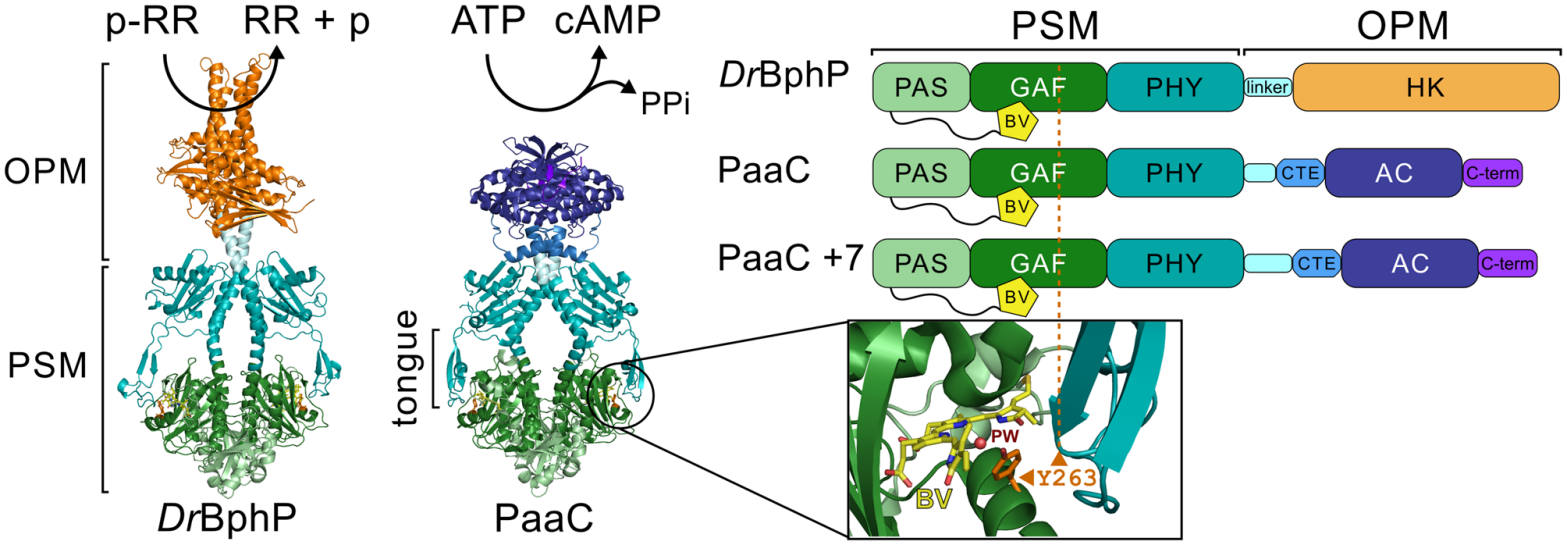
The structures of the phytochromes used in the study. The *Deinococcus radiodurans* bacteriophytochrome (*Dr*BphP) photosensory module (PSM) consists of a PAS domain with a preceding N-terminal extension where the biliverdin (BV) is covalently attached, a GAF domain that physically buries the BV cofactor, and the PHY domain that includes a structurally flexible tongue that extends close to the BV binding site. The GAF domain provides a highly conserved tyrosine residue (amino acid 263 according to *Dr*BphP numbering), which is highlighted in the close-up of the binding site to show its interaction with the cofactor, the central pyrrole water (PW), and the tongue element. The function of the photoreceptor is defined by the output module (OPM) that is linked to the PSM via a linker region. In wild-type *Dr*BphP, this is a histidine kinase (HK). Replacing the HK domain with an adenylate/guanylate cyclase (AC/GC) from *Synechocystis* sp. including its cognate cyclase transducer element (CTE) created functional chimeras with PaaC and the 7 residue longer linker variant PaaC +7 providing red light regulated cyclase activity [16]. The full-length *Dr*BphP structure is based on a homology model from Björling et al. [5], and the PaaC structure is based on the PDB coordinates 6FHT [16]

As far as red light-sensing phytochromes are concerned, these are typically multi-domain sensor systems with a variety of linked output functionalities across different species [1, 23, 48]. The reactions of phytochromes start with the photoisomerization of a covalently bound bilin molecule inside the chromophore-binding PAS (Per/Arnt/Sim) and GAF (cGMP phosphodiesterase/adenylyl cyclase/FhlA) domains (Fig. 1) [64]. For canonical Group I phytochromes [46], photoactivation initiates structural changes in the photosensory module (PSM), consisting of PAS, GAF, and PHY (phytochrome specific) domains, including local changes in side chain rotamers around the bilin chromophore [74], the refolding of the N-terminal extension preceding the PAS domain [22], and the refolding of the hairpin extension in the PHY domain, also referred to as the tongue, from $\beta$-sheet (as in Fig. 1) to $\alpha$-helix in the photoactivated state [11, 54, 56]. Phytochromes thereby frequently cycle between a red light absorbing Pr and a far-red light absorbing Pfr state. Because of the initial bilin isomerization from *ZZZssa* conformation in Pr to *ZZEssa* in Pfr [63, 70] and the coupled rearrangements in local hydrogen-bonding interactions, the photostates can be fairly simply tracked with UV–Vis spectroscopy (Fig. S1). Eventually, the structural changes in the PSM enable the regulation of a variety of biochemical activities in diverse output modules, such as enzymatic activities or biomolecular interactions [23, 74].

In plants, phytochromes regulate a diversity of key processes related to their growth and development, such as shade avoidance and seed germination [36, 47]. Phytochromes exist also in prokaryotic cells, including photosynthetic and non-photosynthetic bacteria, cyanobacteria and algae, as well as in fungi [4, 6, 27, 46]. Due to their less complex domain architecture and simpler requirements for cofactor maturation, a lot of structural and functional studies have recently addressed biliverdin-binding bacteriophytochromes. Even with this subgroup of phytochromes it has, however, been challenging to link atomic-level structural information of both photostates to their corresponding biochemical activities. In some cases, like with *Agrobacterium fabrum* phytochrome (Agp) [4, 30, 35], activity assays have been available but structural information has been lacking. In other cases, like with *Deinococcus radiodurans* bacteriophytochrome (*Dr*BphP) [11, 56], structural information has been available, even if only for the PSM alone, but the enzymatic readout has been missing. Furthermore, the dynamic nature of the underlying signaling processes has been overlooked or its influence underestimated. To date, these shortcomings start to alleviate as structural information for Agp becomes available [53], enzymatic output for the *Dr*BphP has been revealed [73], and the dynamic perspective of the protein subunits in their photostationary states has been reported [24, 51].

Reports of atomic structures for wild-type bacteriophytochoromes including the histidine kinase output modules have been rare, mainly relying on homology models [5], but full-length structural studies of bacteriophytochromes with other types of output modules have been reported [3, 44, 43]. Full-length structural data could be also obtained for a system, where the PSM is naturally linked to a diguanylate cyclase effector domain, allowing to correlate structural changes of the protein with a direct linkage to the enzymatic activity [21]. In the latter case, even structural rearrangements propagating all the way from the cofactor environment through the sensor-effector linker to the active site of the OPM could be described [22]. With respect to synthetic constructs, a guanylate cyclase (GC) from *Synechocystis* sp. including its cyclase transducer element (CTE) was functionally linked to the PSM of *Dr*BphP system creating a phytochrome activated guanylate cyclase that can easily be switched to an adenylate specific cyclase (PaaC), rendering it a versatile optogenetic tool [16]. Functional properties of this synthetic fusion and its linker region could be correlated with structural details of its full-length structure to provide a detailed picture of its signal transduction mechanism [16].

As indicated above, for *Dr*BphP, not only are structural data available for both Pr and Pfr states [11, 56], but recently also functional insights have been enriched [73]. Mutagenesis of critical amino acids has been performed on a large scale [37, 38, 50, 66], and the PSM has been successfully employed for the generation of artificial optogenetic tools [12, 16, 20, 29, 76]. Still, unexpected insights can be observed. For example, the substitution of the tyrosine at position 263 with a phenylalanine [60], resulting in uncoupling of the tongue and chromophore interactions, thus allowing structural properties of Pfr, such as the $\alpha$-helical tongue to be observed in an otherwise Pr-state biliverdin environment. Importantly, the effect of the Y263F substitution was in part correlated with an altered water coordination in the BV environment, a property that could directly be linked to differences in protonation of the cofactor in various photocycle states [9, 33, 50, 60]. With two different enzymatic readouts, coupled to the same kind of PSM, at hand [16, 73], we set out to study in detail the effect of this Y263F substitution in the context of full-length proteins (Fig. 1).

To that end, we studied the relation between the dynamics of the tongue and the output module by means of solvent accessibility of the chromophore [51], FTIR spectroscopy, and biochemical activity assays. In our studies, we used full-length *Dr*BphP and its fragment lacking the output module (*Dr*PSM), as well as an artificial variant with an adenylate cyclase output module and two different functional sensor-effector linker lengths (PaaC and PaaC +7) [16]. Based on the combination of these four variants and their different output module compositions, we show that the output module stabilizes the conformation of the tongue in the dark state, in which it has been shown to switch between folded ($\beta$-sheet) and random coil conformations [24, 51]. With all phytochrome variants and their Y263F mutants we show that the uncoupling between the tongue and the chromophore environment decreases the dynamic range of the enzyme, i.e., the ratio of the enzymatic activities in the dark-adapted and in the light-activated signaling states [76].

Eventually, these insight are helpful for understanding central properties of light-regulated systems, such as the dynamic range of activation. Tunability of the dynamic range over several orders of magnitude and the favorable tissue penetration of red light could enable more advanced optogenetic tools for mammalian systems [72]. Therefore, understanding the relation between biliverdin conformation, chromophore environment and structural, as well as dynamic alterations of the overall structure to regulate the activity of the output domain, is crucial for an improved understanding of the underlying molecular mechanisms.

## Materials and methods

### Protein expression and purification

#### *Dr*BphP variants

The expression plasmids coding for wild-type *Dr*BphP fragments (*Dr*PSM with amino acids 1–502, full-length with amino acids 1–755) were kindly provided by the laboratories of Prof. R. D. Vierstra and Prof. K. T. Forest [10, 64, 65]. The point mutations (Y263F) were introduced by QuikChange Lightning Multi Site-Directed Mutagenesis Kit (Agilent Technologies) and confirmed by sequencing. The (His)$_6$-tagged constructs were expressed in *Escherichia coli* BL21(DE3) as described previously [58]. Purification was performed by means of affinity chromatography followed by size-exclusion chromatography in 30 mM Tris pH 8.0, as described in Ihalainen et al. [28]

#### PaaC variants

Genes encoding for the uncoupled variants of PaaC and PaaC +7 were created by mutagenesis PCR of the codon required for the *Dr*BphP Y263F amino acid exchange in the pETM-11-based vectors of PaaC and PaaC +7 [16] according to the procedure of Liu and Naismith [39]. The primers for the codon optimized sequences are presented in Table S1. The resulting plasmids were sequence verified and transformed in *E. coli* BL21 (DE3) *cya*^-^ pT7Ho1 competent cells for plate screenings and *E. coli* BL21 (DE3) pT7Ho1 for protein expression and purifcation as described previously [16]. The proteins were stored and studied in 10 mM Tris (pH 8.0), 150 mM NaCl, and 2 mM MgCl$_2$.

### Activity assays

#### Phosphatase PhosTag activity assay

The PhosTag activity assay was performed as in [73]. The *Dr*BphP WT and *Dr*BphP Y263F were introduced with pre-phosphorylated response regulator (RR) from *D. radiodurans*. The phosphorylation of the RR was conducted at +37$^\circ$C for 2.8 mg/ml of RR in the presence of 100 mM acetyl phosphate. For the gels in darkness and red light conditions, 0.4 mg/ml and 0.06 mg/ml of phytochrome concentrations were used, respectively. The samples were pre-illuminated with saturating red LED (660 nm, 5 min, on average 13 mW/cm$^2$) or far-red laser ($>780$ nm, Thorlabs, 20 s, 80 mW/cm$^2$) to reach the Pfr or Pr state, respectively during a 5-min incubation at +25$^\circ$C. The dephosphorylation reactions were initiated with 1 mM ATP and the samples were incubated either under red light (as above) or in the dark at +25$^\circ$C during the reaction, and stopped at corresponding time point with 5x SDS loading buffer. For the mobility shift detection of phosphorylated RR (p-RR) proteins, Zn$^{2+}$-Phos-tag® SDS-PAGE assay (Wako Chemicals) was applied. The reactions were run in the 9% SDS-PAGE gels containing 20 $\mu$M PhosTag acrylamide at room temperature with 40 mA according to the instructions of the manufacturer.

To determine the concentration of the p-RR and the rate of the spontaneous breakage of p-RR to RR, a separate control gel without phytochrome samples was run otherwise as described above. First, the concentration of the p-RR from the RR/p-RR mixture was quantified from their intensity ratio, obtained by ImageJ program, revealing a 50% phosphorylation rate (Fig. S2A). Second, a time series with *Dr*BphP WT and Y263F, where the reactions were kept at +25$^\circ$C for different times (otherwise on ice), were made. The band intensities were normalized to the zero time point and fitted to a single polynomial function. The slope was used to correct the activity assay gel results to investigate the phytochrome activity alone (Fig. S2B).

The reaction catalyzed by *Dr*BphP WT and Y263F at each time point was followed by plotting the p-RR and phytochrome concentration ratio at each time point. The gels for dark and red light conditions were both repeated three times (Fig. S3) from which the value for each time point was averaged, and errors as standard deviation were calculated. The reaction is assumed to proceed as in the following equation:

1 $$\begin{aligned} {[}E] + [S] \underset{k_{-1}}{{\mathop {\rightleftharpoons }\limits ^{k_{1}}}} [ES] \xrightarrow {{k}_{2}} [E]+[P]. \end{aligned}$$

Assuming that the k$_2$ is the rate limiting step of the reaction, the first four time points of the reaction were fitted to a linear function to derive the initial velocities for reactions in dark and red light conditions for *Dr*BphP WT and Y263F (Fig. S4).

#### Adenylate cyclase activity assays

Adenylate cyclase activity assays were performed according to the procedure described in [16]. Briefly, reactions were set up using 20 $\mu$M PaaC and PaaC +7 at a substrate concentration of 1 mM ATP at 20$^\circ$C. For light state measurements, samples were pre-illuminated for 10 min using a 660 nm LED (Thorlabs) at 34 mW/cm$^2$. Dark state measurements were performed under non-actinic conditions with indirect dim green light. After various incubation times, reactions were stopped by heat inactivation at 95$^\circ$C for 1 min. The soluble part was subsequently analyzed by HPLC and substrate and product concentrations quantified. Measurements were done for various time points and initial velocities were derived from linear fits weighted by the standard deviation of three independent replicates.

The plate screening assay for adenylate cyclase activity was performed as described in [16]. Briefly, *E. coli* BL21 (DE3) *cya*^- ^ cells containing the helper plasmid pT7Ho1 were transformed with plasmids encoding the adenylate cyclase variants. As a negative control, a pETM-11 plasmid with a gene encoding for a non-cyclase blue light-sensitive photoreceptor protein (AppA$\Delta$C) was transformed [67]. Cells were grown in LB medium supplemented with 0.36% glucose at 37$^\circ$C. An aliquot was then harvested, the cells collected by centrifugation and resuspended to reach an OD600 of 20. 4 $\upmu$l of the cell suspension were spotted on LB agar plates [10 $\upmu$M IPTG, 60 $\upmu$g/ml 5-bromo-4-chloro-3-indolyl-$\beta$-D-galactopyranoside (X-Gal), 10 $\upmu$g/ml 5-aminolevulinic acid, antibiotics] and incubated in the dark or under constant red light illumination for several hours at 37$^\circ$C, until a change in coloration of the colonies was observed.

### Steady-state FTIR spectroscopy

By illuminating the sample with saturating 661 nm or 775 nm LED, providing Pfr or Pr state, respectively, the difference FTIR-signal between the states was measured using a Nicolet Magna IR-760 FTIR spectrometer as described in Kurttila et al. [34]. The procedure was repeated for H$_2$O diluted and D$_2$O diluted 30 mM Tris buffers at pH 8.0. The difference spectra were normalized to the negative biliverdin D-ring C=O stretch band [18] at 1712 cm$^{-1}$ (in H$_2$O) or 1700 cm$^{-1}$ (in D$_2$O).

### pH jump with stopped-flow

The so called pH-jump experiment records kinetic changes of absorption properties of the phytochrome after rapid pH change of the solution. The method is adapted from Rumfeldt et al. [51] However, here the aim was to record absorbance changes in the sub-second time-scale. Therefore, a TgK Scientific Hi-Tech KinetAsyst Stopped-Flow system that has a dead time of 1–2 ms (instead of 13 s with manual mixing) was used.

The phytochrome samples were freshly prepared in 0.4 mM Tris pH 8.0 with an absorbance of about 0.4 at 700 nm. The stopped-flow device was used to rapidly mix 60 mM glycine pH 10.8 buffer with the initially prepared phytochrome mix, resulting in pH 10.8 in 30 mM glycine, 0.2 mM Tris buffer concentrations. The absorbance changes were detected with an integration time of 1.6 ms and low light intensity to minimize the photoconversion during the early time points. Control experiments in 15.4 mM Tris at pH 8.0 confirmed that the fast phase does not suffer from detection driven photoconversion. All the measurements were repeated at least 15 times, including measurements from separate sample batches, and with time intervals varying between 8 ms, 15 ms, 80 ms and 150 ms. All the measurements were averaged to reach an adequate signal-to-noise ratio. The measurements were carried out at 21$^\circ$C.

The “slow phase” of the pH-jump measurement to determine the end point absorption was measured using manual mixing and a Cary 8454 UV–Vis spectrometer (Agilent Technologies) as described in [51] with the data analysis, described earlier.

The fast phase required a bi-exponential fitting function according to the following equation:

2 $$\begin{aligned} A_{\text {measured}} = \textit{A}_1 \times \textit{e}^{-k_1t} + \textit{A}_2 \times \textit{e}^{-k_2t} + A_{\text {fast endpoint}}, \end{aligned}$$

where *A*$_{\text {measured}}$ is the normalized absorbance measured at 700 nm, *A*$_i$ and *k*$_i$ are the amplitudes and rate constants for the fast phase, respectively, *t* is time and *A*$_{\text {fast endpoint}}$ is the final absorbance value of the fast phase. Further, the data were scaled in respect of the total change according to the final endpoint of the whole process.

### Natural diversity in bacteriophytochrome sequence space and coevolution analysis

During characterization of sequence space of the PadC family [7], one family member was identified that naturally features F at the position corresponding to Y263 in *Dr*BphP. HHBlits searches with this sequence revealed several homologs featuring identical sensor-effector linker lengths with the same substitution. In addition, residues identified by Gremlin coevolution analysis [45] to correlate with the Y263 position [7] show interesting substitutions in this subfamily that indicate a different biliverdin environment, especially around the D-ring.

**Fig. 2:**
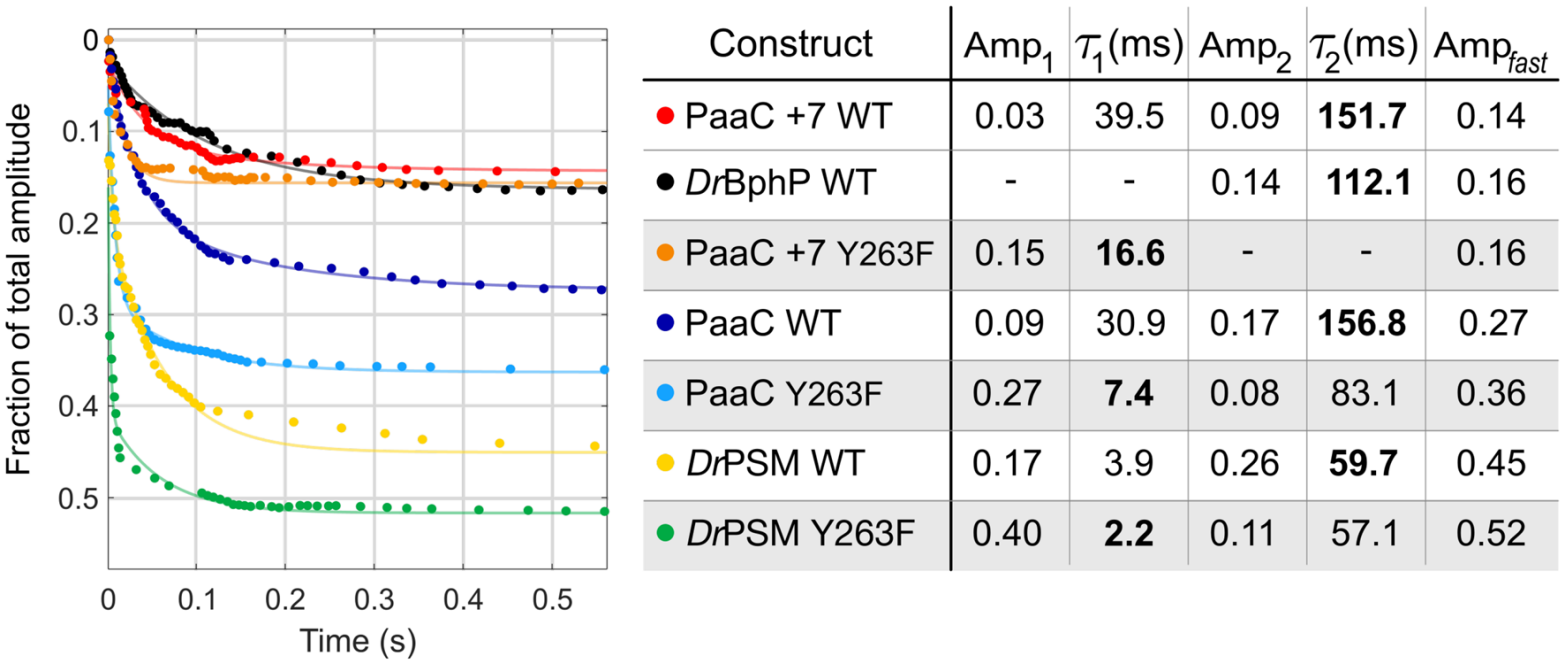
The BV deprotonation, followed at A$_{700}$ as a function of time after a rapid pH jump from pH 8.0 to 10.8. The data (dots) is normalized to the A$_{700}$ at pH 8.0, fitted to a double exponent function Eq. 2 (solid line), and scaled to the total amplitude of the whole reaction, shown in Fig. S5. The fitted time constants and their amplitudes are presented in the table on the left side. The more dominating fractions of the fits are marked in bold. The Y263F mutants are highlighted with grey. The sum of squared errors (SSE) of the fits are $<0.0067$ for *Dr*PSM and $<0.0027$ for the full-length systems

## Results and discussion

### The output module stabilizes the “closed” conformation of the tongue in its resting state

We recently introduced a method where solvent access to the chromophore-binding pocket can be studied by a quick pH jump [51]. This allows determination of the tongue fluctuation between “closed” and “open” conformations in the Pr state. In *Dr*BphP, the closed tongue conformation protects the BV environment from solvent and the open conformation allows solvent access to BV resulting in deprotonation of the cofactor at high pH ($>10.5$). Deprotonation of BV decreases its absorption at the phytochrome Q-band (around 700 nm) (Fig. S1) [26, 61], and in terms of kinetics of the absorbance changes, two phases can be observed (Fig. S5). In the population responsible for the fast phase, the tongue is in the “open” conformation and BV thus deprotonates rapidly. The slow phase corresponds to the kinetics of tongue opening from its closed state [51].

The use of stopped-flow spectrophotometry enabled measuring absorbance changes on sub-second time-scales (Fig. 2). In most of the studied samples, the resulting kinetics required a minimum of two-exponential fits. We note that the kinetic signal in the range around 20 and 80 ms is challenging to fit with exponential functions. The signal in this time range probably reflects complex deprotonation mechanics in the studied systems. However, our major aim was to find out the dynamic nature of the tongue system. In all cases, the total amplitude of the decay (Amp$_{\text {fast}}$ in Fig. 2) corresponds to the population of the open conformation present at the initiation of the jump. If the tongue is in a conformation that allows solvent access to the BV environment, deprotonation takes place essentially on the time-scale of 150 ms. To reveal the fraction of the fast phase amplitude relative to the total absorbance change, we also recorded the overall absorbance decrease (Fig. S5). The smallest fast phase amplitudes are observed in PaaC +7 (0.14), *Dr*BphP WT (0.16) and PaaC +7 Y263F (0.16). These numbers imply that in the dark state, 86% and 84% of the population, respectively, have the tongue in a “closed” conformation, i.e., folded as a $\beta$-sheet thereby preventing solvent access to biliverdin. The shorter the linker length is between PHY and OPM (PaaC vs. PaaC +7 and *Dr*BphP) or if the OPM is completely missing, the greater the fraction of the fast phase amplitude that is observed, up to values of 0.52 for *Dr*PSM Y263F mutant. This shows that the OPM, the linker region, and the tyrosine side chain in the 263 position stabilize the conformation of the tongue as a $\beta$-sheet in the dark state.

**Fig. 3:**
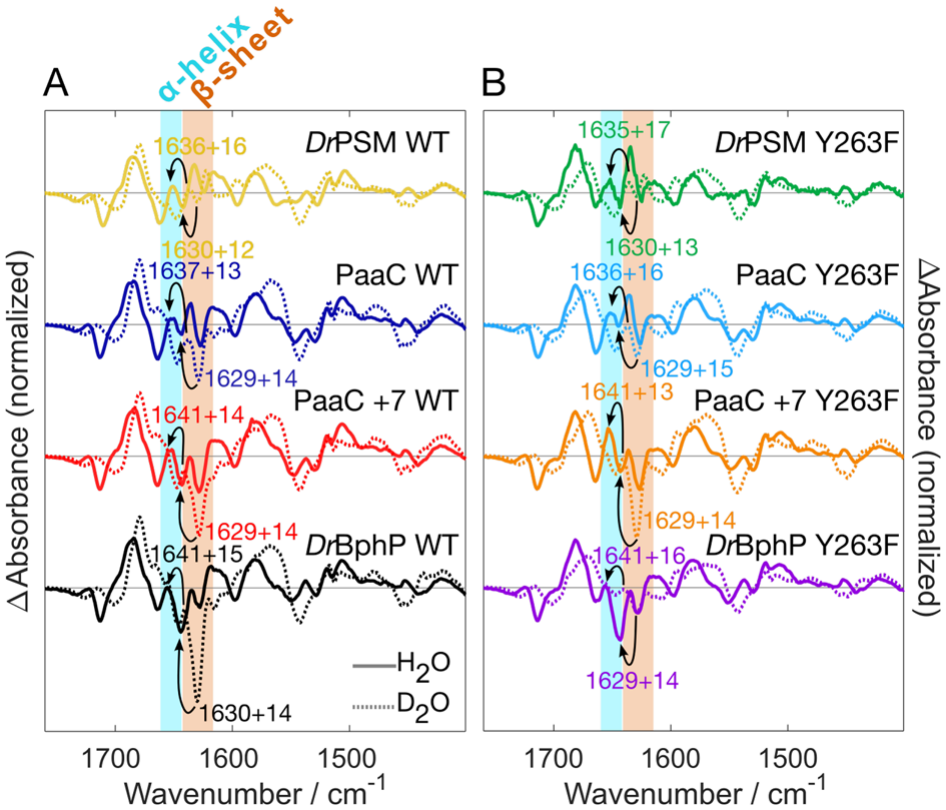
The effect of the output module on the changes in amide I signals detected by FTIR. The difference spectra (Pfr *minus* Pr) were measured in H$_2$O (solid line) and D$_2$O (dotted line). The numbers with arrows indicate the shift from D$_2$O to H$_2$O of the negative signals within the $\beta$-sheet region (1615–1638 cm$^{-1}$, rust background) and the positive signals within the $\alpha$-helix region (1642–1660 cm$^{-1}$, cyan background). **A** *D. radiodurans* photosensory module and all full-length systems without any point mutations, **B** Y263F variants of the same constructs. The H$_2$O and D$_2$O spectra are normalized according to the negative 1712 cm$^{-1}$ peak and D$_2$O spectra are scaled with a factor of 0.5 to match the size of H$_2$O spectra

The Pr to Pfr spectral changes in the mid-IR-region reveal the changes of the biliverdin, its surroundings, and the protein scaffold upon photoactivation [18, 17, 28, 31, 34, 54, 62]. Already 25 years ago, Foerstendorf et al. performed extensive FTIR analysis of the phytochrome proteins, distinguishing the IR-signals from the chromophore from the protein scaffold. However, the link to the structural changes of the protein were hampered due to the lack of crystal structures in those early days [18, 17]. Later on, Stojković et al. determined clear changes in the amide I region between Pr and Pfr states for *Rhodopseudomonas palustris* systems, which they pinpointed to the tongue region [54].

In terms of *Dr*BphP, the secondary structural changes are slightly more difficult to observe [28, 38, 59], albeit the clear changes in the crystal structures [11, 56]. Most likely, this is due to compensating signals from the chromophore binding PAS-GAF domains [59]. Nevertheless, we can detect a clear Pr-related (negative signal in the Pfr *minus* Pr difference spectrum) signal at 1642–1644 cm$^{-1}$ in *Dr*BphP in H$_2$O, which is down shifted to 1629–1630 cm$^{-1}$ and better revealed in respect to intensity of other signals in D$_2$O (Fig. 3). We relate this signal to the disappearance of the $\beta$-sheet structure upon the photoconversion to the Pfr state [9, 32, 54, 60, 68]. The positive appearance of the $\alpha$-helix in the Pfr-state is somewhat buried under other signals of the system but a small positive contribution at 1635–1641 cm$^{-1}$ and 1650–1657 cm$^{-1}$ in D$_2$O and H$_2$O, respectively, can be observed.

The constructs in our study reveal variation of the size of the negative signal ($\beta$-sheet in Pr) (Fig. 3A). The signal decreases in PaaC WT in comparison to *Dr*BphP WT, and is the smallest in *Dr*PSM. Relatively, the PaaC +7 has slightly bigger negative signal with respect to the PaaC. As this signal can be rather safely related to the changes in the tongue region, these observations correlate with the solvent accessibility study shown above: a shorter sensor-effector linker or a completely missing OPM results in a more flexible tongue region. Naturally, we can not rule out compensating effects of other signals among the different constructs.

Despite the lack of the prominent FTIR difference signal indicative of $\beta$-sheet to $\alpha$-helix transition and the challenging deprotonation kinetics in the stopped-flow data, our results are in good agreement and support each other. The solvent accessibility phenomenon with pH-jump method has been studied so far only in *Dr*BphP [51]. However, in various prokaryotic phytochrome crystal structures, for example in *Rp*BphP2 [71], *Is*PadC [21], *Sa*BphP1 [75] and Cph1 [15], the $\beta$-folded tongue appears to block the solvent access to the chromophore-binding pocket, like in *Dr*BphP. Therefore, it would be interesting to see, if the solvent access is similar for example in *Rp*BphP2 or Cph1, that show relatively large $\beta$-sheet to $\alpha$-helix transition in their FTIR difference spectra [54, 62]. On the other hand, some crystal structures of plant phytochromes, like *Gm*PhyB [42] and *At*PhyB [10], suggest that the tongue allows solvent access to the chromophore even in the Pr state. Therefore, the pH-jump method would help to verify this observation in solution.

**Fig. 4:**
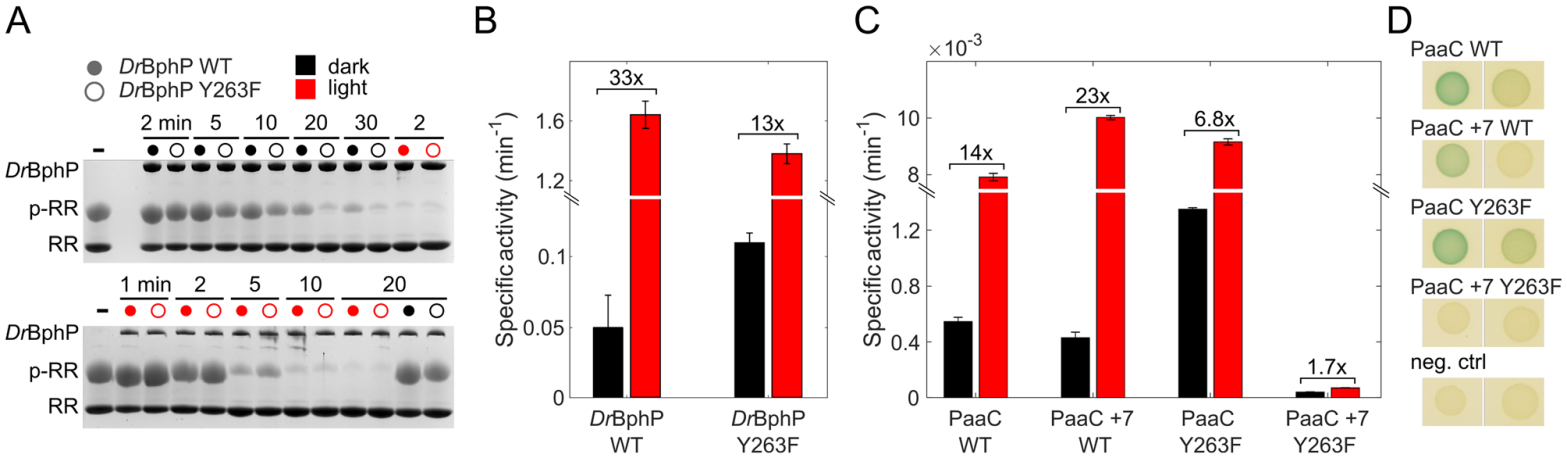
Phosphatase and cyclase activity of the phytochrome variants in dark and red light. **A** The declining amount of phosphorylated response regulator (p-RR) upon phosphatase activity was followed at different time points in the dark (top panel) and under red light (bottom) for *Dr*BphP WT and Y263F with a PhosTag assay. The phytochrome (enzyme) concentration used in the bottom gel is 6 times less than on the top. **B** The specific activity of *Dr*BphP WT and Y263F in dark and red light were determined from the gels in A (the kinetic plots in Fig. S4). A y-axis break was introduced between 0.14 and 1.1 min$^{-1}$ for better visualization of the dark state activity. The numbers on top indicate the dynamic range for *Dr*BphP WT and Y263F. **C** Specific activities of PaaC variants. Black bars correspond to dark state activities, while red bars reflect specific activities in the illuminated states. A y-axis break was introduced between 0.0015 and 0.0075 min$^{-1}$ to enable better visualization of the low PaaC +7 Y263F activity. **D** Representation of adenylate cyclase screening of the PaaC variants in cyclase deficient *E. coli* cells, grown at 37 $^\circ$C

### Tongue flexibility affects the biochemical activity of phytochromes

The flexibility of the tongue region may also relate to the biochemical activity of the constructs. *Dr*BphP functions as a phosphatase and its activity can be switched on with red light [73]. Also, the cyclase activity of the PaaC variants switches on with red light [16]. Thus, in the dark state, *Dr*BphP and the PaaC variants should, in principle, be inactive. In spite of this, the dynamic tongue fluctuates between ‘closed’ and ‘open’ conformations in the dark state [24, 51]. To understand the consequence of tongue dynamics on the activity, we ran an activity assays of *Dr*BphP and PaaC variants (Fig. 4), and the switching between Pr and Pfr states was confirmed with UV–Vis spectroscopy (Fig. S1). The phosphatase activity of *Dr*BphP was followed by tracking the disappearance of the substrate p-RR with a PhosTag activity assay [73] in dark and red light conditions (Fig. 4A, all gels available in SI Fig. S3), taking into account the spontaneous breakage of p-RR (Fig. S2). The specific activities of *Dr*BphP in dark and red light conditions, derived from the initial velocity of the reactions (Fig. S4), reveal that *Dr*BphP is not completely inactive in the dark state (Fig. 4B). In the dark, *Dr*BphP WT has specific activity of 0.050 min$^{-1}$. Under red light, the specific activity increases to 1.65 min$^{-1}$, 33 times higher than in darkness. The ratio between the red light and dark state-specific activities corresponds to the dynamic range of the system.

The specific activities of the PaaC variants reveal that also all of them have some dark state activity (Fig. 4C). Here, the same output activity allows the comparison between PaaC and PaaC +7 revealing that the specific dark state activity in PaaC (0.5*10$^{-3}$ min$^{-1}$) is higher than in PaaC +7 (0.4*10$^{-3}$ min$^{-1}$). This correlates with the more dynamic tongue region in PaaC compared to PaaC +7 as seen in the pH-jump and FTIR experiments (Figs. 2 and 3A). The lower dark state activity in PaaC +7 WT in comparison to PaaC WT is also visible in the cell-based screening, where green color indicates enzymatic activity of the PaaC variants (Fig. 4D). The introduction of the additional turn in the linker region in PaaC +7 WT increases the dynamic range also by increasing the specific activity under red light from (8*10$^{-3}$ min$^{-1}$) to (10*10$^{-3}$ min$^{-1}$) (Fig. 4C). As a result, the fold change in PaaC +7 WT is 23-fold, which is 64% higher than the 14-fold change in PaaC WT.

Due to the different chemical outputs, the specific activities between *Dr*BphP and PaaC variants are difficult to compare. Still, the dynamic ranges are comparable (Fig. 4B, C). They show that the natural system *Dr*BphP has the highest fold change (33-fold). The artificial PaaC variants do not reach the dynamic range of the natural system with 23-fold and 14-fold changes in PaaC +7 WT and PaaC WT, respectively, but they still operate at comparable levels. We note that none of the systems is completely inactive in the dark in vitro. This suggests that organisms must have developed coupled regulatory mechanisms to control the on and/or off states of signaling components.

**Fig. 5:**
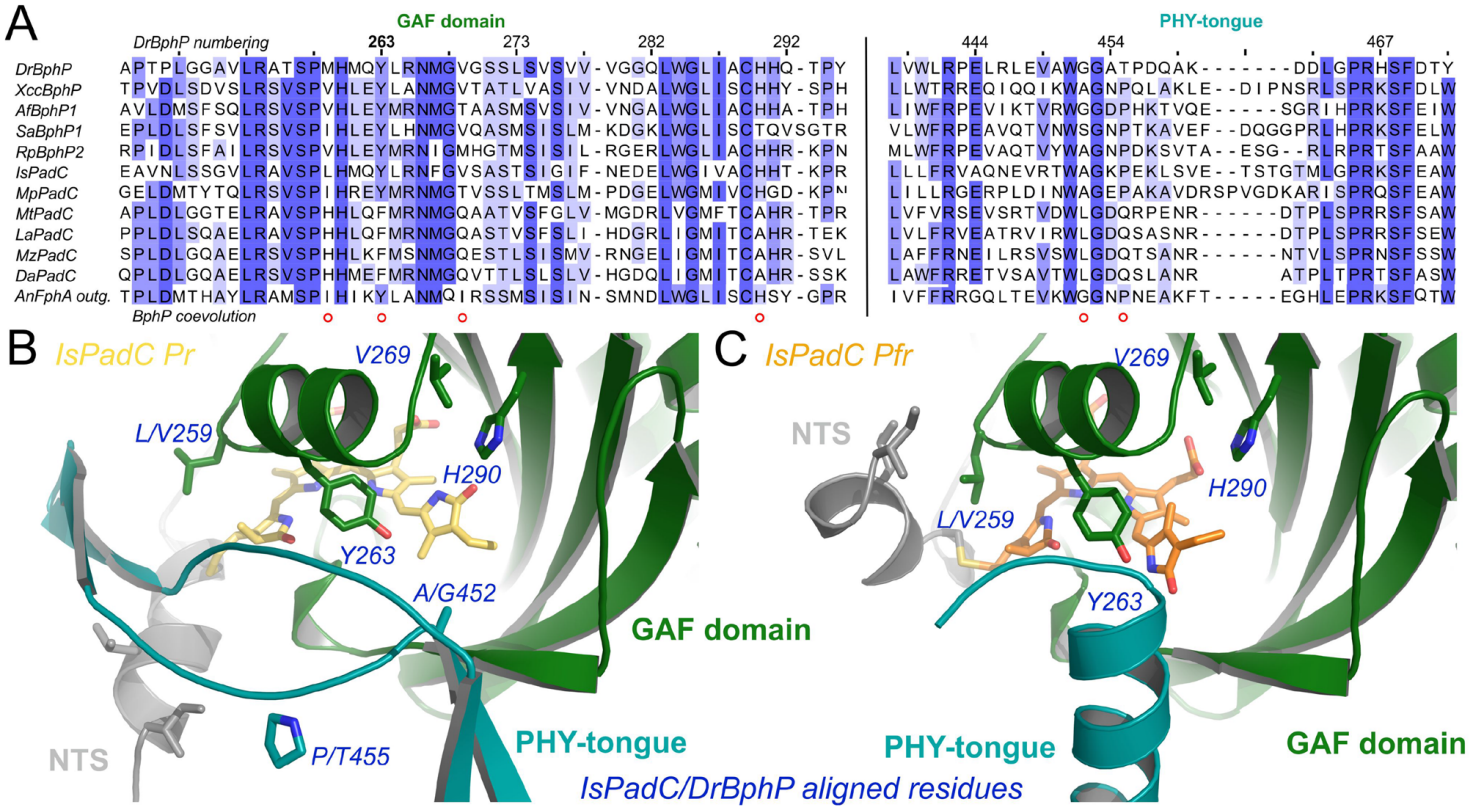
Sequence conservation analysis of some well studied bacteriophytochromes compared to the subfamily of PadCs with a naturally occurring Y263F substitution. **A** Sequence alignment of two amino acid stretches involved in BV D-ring coordination. The fungal FphA outgroup is shown at the bottom of the alignment. The other sequences from top to bottom correspond to *Dr*BphP, *Xanthomonas campestris* BphP, *A. fabrum* BphP1 (Agp1), *Stigmatella aurantiaca* BphP1, *Rs. palustris* BphP2, *Idiomarina* species A28L Phytochrome activated diguanylate cyclase (PadC), *Marinobacter persicus* PadC, *Microbacterium trichothecenolyticum* PadC, *Leifsonia aquatica* PadC, *Mycetocola zhadangensis* PadC and *Demequina aestuarii* PadC. **B**+**C** Close ups of the three-dimensional structures corresponding to the closest homolog structure available for the PadC family with the natural Y263F substitution—*Is*PadC—in Pr [21] and Pfr conformations [22], respectively. The BV cofactor is shown in yellow and orange, respectively. Colors of individual domain elements are the same as used in Figure 1. Numbering correspond to *Dr*BphP

### Influence of the Y263F mutation on the tongue dynamics and further to the enzymatic activity

Above, we have shown that the full-length linker and OPM strengthen the stability of the tongue, which also influences the dynamic range of the enzyme activity. Previously, the effect of the uncoupling between the chromophore and the tongue by the Y263F mutation has been demonstrated in structural terms in *Dr*BphP PSM [60]. Now, we link the uncoupling effect to the tongue dynamics and enzymatic activity in full-length *Dr*BphP and PaaC variants. The activity assays of *Dr*BphP and PaaC WT and Y263F mutants show that the Y263F mutation increases the dark state activity (Fig. 4B, C). In *Dr*BphP Y263F and PaaC Y263F, the dark state activity is 2 and 2.5 times higher, respectively, than in their respective WT. The red light activity is slightly lower in *Dr*BphP Y263F and slightly higher in PaaC Y263F than in their WT counterparts. However, the evident decrease of the dynamic range in both *Dr*BphP and PaaC appears to be mainly due to the increase of the dark state activity in the Y263F mutants.

Interestingly, almost negligible cyclase activity for PaaC +7 Y263F was revealed. Compared to the fully functional PaaC +7 construct, which has a relatively high dynamic range, the light state activity is approximately 140 times smaller in PaaC +7 Y263F (Fig. 4C). However, there is still a slight difference in activity between dark and red light conditions. The results of the in vitro activity assay are to some extend repeated in vivo, (Fig. 4D). Both PaaC and PaaC Y263F have green color in the light state, and some dark state activity when grown at 37 $^\circ$. PaaC +7 also shows light state activity, but not with the same intensity as PaaC and PaaC Y263F. PaaC +7 Y263F shuts down also in vivo and no activity is observed in dark or light conditions.

The FTIR spectra show that the Y263F mutation decreases the intensity of the negative Pr-related $\beta$-sheet signal in PaaC and *Dr*BphP in comparison to WT, seen in particular in D$_2$O measurement (Fig. 3). In PaaC WT and PaaC +7 WT the $\beta$-sheet signal in H$_2$O is bigger in comparison to their Y263F mutants, but in *Dr*BphP WT it is smaller than in its Y263F variant, most probably due to overlapping signals. In contrast, in D$_2$O, the PaaC +7 Y263F presents slightly increased signal intensity in comparison to the WT. This might indicate a stabilized tongue architecture even in the Y263F variant due to the more stabilized coiled-coil effector of this system, which would also correspond to the decreased overall activity. The typically decreased refolding of the tongue in the Y263F variant has been shown earlier in *Dr*PSM, discussed together with other spectral changes caused by the mutation [60].

The uncoupling of the chromophore and the tongue due to the Y263F mutation affects the solvent accessibility, as expected. In all Y263F mutants, the amplitude of the fast change due to deprotonation is bigger than in their WT counterparts (Fig. 2) meaning that due to uncoupling, the solvent accessibility to the chromophore is increased. This has been showed before in the photosensory module [51] and here also in full-length variants (Fig. 2). The increased tongue dynamics are observed in FTIR as well as solvent accessibility studies, and we connect these fluctuations to the increased dark state enzymatic activity (Fig. 4B, C). In PaaC +7 WT vs. Y263F counterparts, the difference in the tongue dynamics is rather small, observed both by FTIR (Fig. 3) and the solvent accessibility. Nevertheless, this variant features a rather inhibited state, which correlates with a stabilized Pr tongue conformation (Fig. 4).

A closer look at the fast phase time constants of the solvent accessibility studies reveals a difference between the Y263F mutants and their wild-type counterparts (Fig. 2). In all Y263F variants, the dominating amplitude has a time constant of 10 ms. When the hydroxyl group at residue 263 is present, the time constant of the dominating component is around 100 ms. The difference between dominating time constants is observed even in both PaaC +7 s, although the total amplitude remains similar despite the Y263F mutation. This suggests that the Y263 has a role in the BV (de)protonation mechanism, possibly through water network interactions [13]. The sub-second deprotonation data possibly include information about the mechanism as the bi-exponential fit is not sufficient to capture all the features of the data. However, these data alone are inadequate to make interpretations about the (de)protonation mechanism in detail and will not be discussed further in this paper, but should be studied more carefully in the future.

**Fig. 6:**
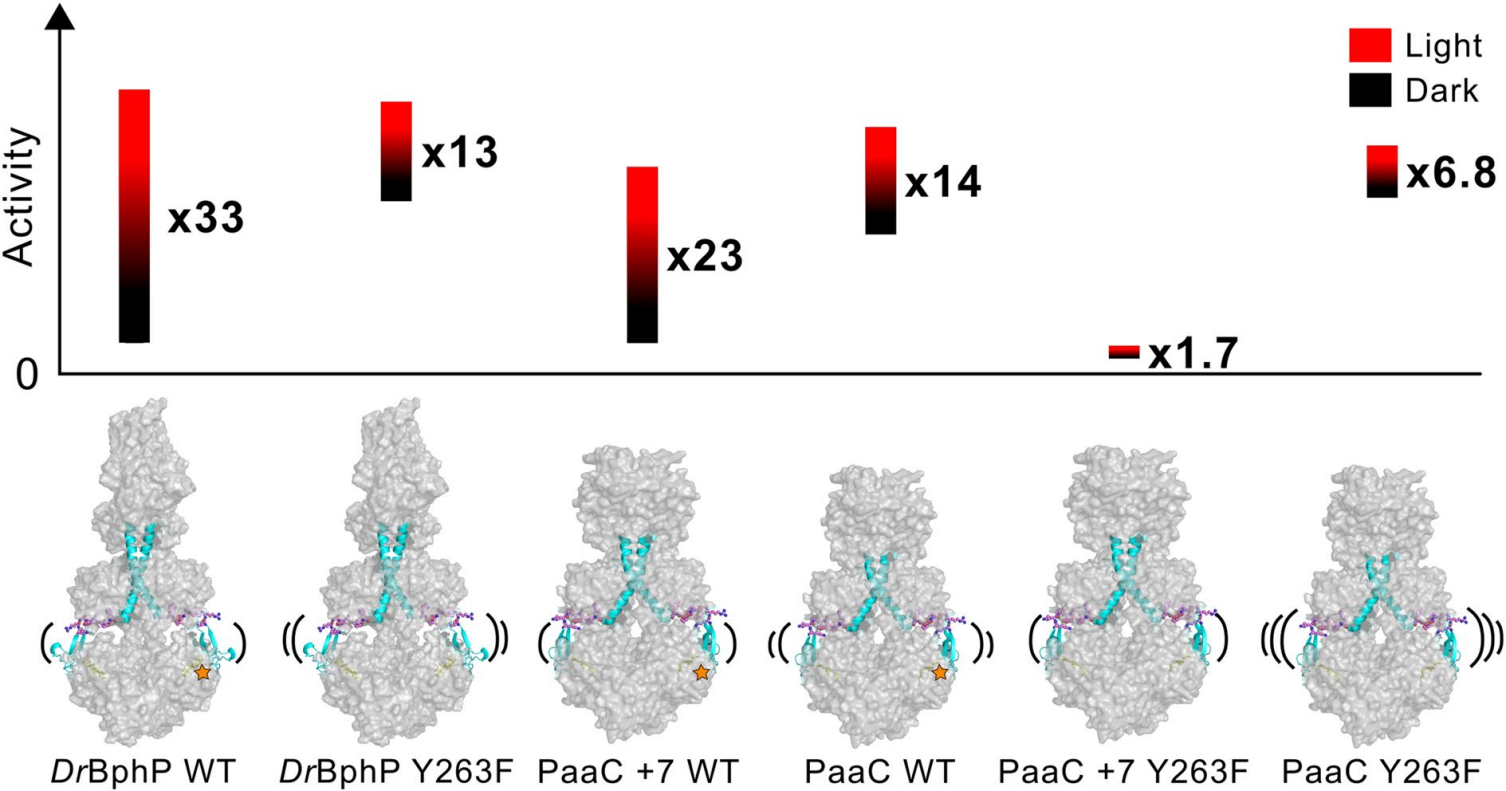
Overview of dynamic ranges of *Dr*BphP and PaaC variants. The height of the bars represents the dynamic range of each construct, and the amount of activity in the dark or under red light is illustrated as the relative position of the bars on the y-axis in relation to each other. We interpret the changes in dynamic ranges either by (1) different output modules with increasing/decreasing cross-talk between the linker and the tongue or (2) by the uncoupling between the chromophore, tongue and thus output module due to the Y263F substitution. The tongue and the N-terminal PHY-helix that finally extends into the sensor-effector linker helix are directly connected *via* eight residue long chains, shown with ball-and-stick model in purple. The dynamics of the tongue are illustrated in the structures, and the presence of the coupling hydroxyl group at position 263 is marked with a star. The structure of the PaaC +7 variants is an illustration based on the PaaC structure with additional turns in the linker helix (cyan)

### The linkage of the tyrosine at 263 to other structural elements in bacteriophytochromes—coevolution alignment

Above, we demonstrated the influence of Y263 on tongue dynamics as well as for enzymatic activity in *Dr*BphP-based systems. The importance of tyrosine at this position can also be demonstrated by a small sequence alignment-coevolution study. While the vast majority of bacteriophytochromes, but also more distantly related phytochromes such as the fungal outgroup of the alignment (Fig. 5), show a strictly conserved tyrosine at this position, a small subfamily of bacteriophytochromes naturally features a replacement of tyrosine with phenylalanine Y/F263 (Fig. 5). Interestingly, members of this family show concomitant changes at other critical biliverdin-binding residues (red circles in Fig. 5). Apparently, different BV environments can fulfill similar functions for the stabilization of the intricate rearrangements accompanying light activation. Since these compensating mutations are not available in our Y263F variants, the apparently important regulatory network in the D-ring environment is missing and, therefore, negatively affects the dynamic ranges of the variants.

As shown above and in [60], the Y263F variant in *Dr*BphP causes uncoupling of the chromophore and the tongue and decreases the photoconversion efficiency [60]. Apparently, the productive formation of Pfr is reduced by an increased structural heterogeneity of the chromophore in the Pr state, which in turn allows sampling of Pfr-like conformations even in the dark. Along these lines, shorter constructs bearing only the chromophore-binding domain of *Dr*BphP system and featuring the same Y263F substitution have been shown to exhibit increased fluorescent yields, making them interesting candidates as fluorescent reporters [1]. Also for more distantly related bacteriophytochromes from *Rhodopseudomonas palustris* (*Rp*BphP), the analogous amino acid replacement was shown to only moderately affect Pfr formation in the classical Pr-Pfr converting *Rp*BphP2, whereas it completely abolishes Pr to Pnr formation in *Rp*BphP3. Interestingly, the region responsible for the uncommon photocycle characteristics can also be attributed to residues lining the biliverdin D-ring [69]. The most detailed characterization of a system featuring the corresponding Y263F substitution in Cph1 from *Synechocytsis* also revealed interesting parallels, while also showing important differences at a structural level [40]. In Cph1 the substitution of Tyr to Phe also increased the fluorescence yield and slowed down formation of an otherwise typical Pfr spectrum, which was attributed to different populations of structurally heterogeneous biliverdin substates in the Pr state. At a structural level, interesting changes of tongue residues in the region of biliverdin contact sites were also observed. However, it was unclear whether these are due to crystal contacts or due to the introduced amino acid substitution. Overall, the tongue architecture was still in a Pr-like conformation even though some potentially functionally relevant rearrangements of the photosensory module were observed by the changes in the PHY domain orientation relative to the chromophore-binding domain [40].

### A short linker region between the OPM and tongue rationalizes the observed effects on the tongue dynamics and enzymatic activity

One of the main conclusion of our study is that the composition of the OPM influences to the properties of the tongue region. First, the presence of the OPM considerably stabilizes the tongue in its Pr state in comparison to with the PSM only. Both FTIR spectra and pH-jump experiments imply more loose tongue systems in the PSM construct. Secondly, by increasing the stability of the coiling linker helix towards the OPM, the stability of the tongue region increases.

A closer look at the crystal structures reveals an interesting detail about the link between the tongue and the OPM. There are only eight amino acids between the tongue and the helix at the C-terminal end of PHY domain that finally extends as the coiled-coil linker of the OPM, highlighted in purple in Fig. 6. The short linker between the tongue and OPM regions gives an intuitive picture of a direct channel between the structural changes in the tongue region and the properties of the OPM. Consequently, changes in the OPM are easily reflected back to the tongue, and hence in the chromophore-binding pocket (Fig. 6).

In photoreceptors, the enzymatic activity of the OPM can be either increased or suppressed with light [41]. For example, in the case of *A. fabrum* phytochrome, red light suppresses the kinase activity [4, 30, 35, 73]. In nature, the dynamic range in photoreceptors can cover at least three orders of magnitude. For example, a fold change of over 1000-fold has been recorded for the cGMP producing rhodopsinguanylyl cyclase from *Catenaria anguillulae*, where the activity is switched on with green light [52]. The dynamic range of a LOV-domain-based photoswitch has been improved from 5- to 70-folds by rational mutations [55]. In our WT systems, the fold change varied from 14-fold in PaaC WT to 33-fold in *Dr*BphP WT. It has been pointed out by Ziegler and Möglich, that thermodynamically the magnitude of the dynamic range is mainly dependent upon how well the enzymatic activity can be shut off in the non-active state, in our case the dark state [76]. The more dynamic the tongue is, either due to weaker stabilization from the OPM, due to uncoupling between the chromophore and tongue, or due to other properties, the smaller the dynamic range. The cross-talk between the tongue and the OPM seems to be the one of the key tuning nobs of the dynamic range in the phytochromes, and the short linker provides an effective communication wire between them.

## Conclusions

The signal transduction from the light absorbing chromophore to the enzymatically active OPM as well as stabilizing the light state conformation require delicate intramolecular regulation mechanisms. The structural events in bacteriophytochromes can be divided into tiers, where the small scale reactions take place around the chromophore, which are then linked to secondary structural changes of the tongue and the N-terminal segment [23], and finally to large-scale structural changes or to changes in the activity of OPM [23, 74]. Previously, the uncoupling of the different tiers of a phytochrome has been demonstrated in the photosensory module alone by Takala et al. [60]. They showed that the chromophore, the flexible tongue and the overall protein structure do not always act hand in hand. When the linkage between the chromophore and the tongue, a hydroxyl group in Y263F, is removed, even though the chromophore is in its Pr conformation (*ZZZssa*), the tongue and the globular structure can resemble the structures of the Pfr state. Here, we demonstrated the uncoupling phenomenon in terms of enzymatic activity: the same point mutation position (Y263F) in the full-length system leads to a more flexible tongue region, and further, to lower the dynamic range of the enzymes due to increased dark state activity. The results could be rationalized by a larger population of “open” tongue systems in the dark state. This highlights the role of the tyrosine in the position 263 in linking the PHY domain to the chromophore-binding pocket, and thus in the allosteric communication between the sensory and effector domains of *Dr*BphP.

Furthermore, a direct interplay between the OPM, the tongue, and thus the chromophore-binding pocket in a bacteriophytochrome is demonstrated. The composition of the OPM relates directly to the dynamics of the tongue, which again influences the dynamic range in enzymatic activity of the studied samples. With three different phytochrome constructs, wild-type *Dr*BphP, PaaC and PaaC +7 all bearing the same photosensory module from *Dr*BphP, we show that all the output modules stabilize the tongue in comparison to the photosensory module alone, and that the natural *Dr*BphP system operates with the largest dynamic range. However, the artificial red light-responsive adenylate cyclase PaaC +7 does not fall far behind. The reported fold changess of the wild-type systems range from 14-fold to 33-fold, which is promising for optogenetic applications.

The OPM is connected to the tongue, i.e., the hairpin extension, via a helical linker and a short connector between the linker helix and the tongue. All together, these structures and the coupling of the tongue and chromophore assure a stiff-but-controllable structural connection between the enzymatic activity and the photoactivated state of the chromophore, like a two-way street.

## Supplementary Information

Below is the link to the electronic supplementary material.

Supplementary file1 (PDF 17,524 KB)
