# Supplementary material for: The structural effect between the output module and chromophore-binding domain is a two-way street via the hairpin extension
Source: Photochem Photobiol Sci. 2022 Aug 19;21(11):1881–94. doi: 10.1007/s43630-022-00265-5 (PMC9630206; doi:10.1007/s43630-022-00265-5)
Supplement: Supplementary file 1 — Supplementary file1 (PDF 17,524 KB) [file 43630_2022_265_MOESM1_ESM.pdf]

Supporting Information for The structural effect between the output module and chromophore binding domain is a two-way street via the hairpin extension

Moona Kurttila[a], Stefan Etzl[b], Jessica Rumfeldt[a], Heikki Takala[a], Nadine Galler[b], Andreas Winkler[b*] and Janne A. Ihalainen[a**]
[a] *University of Jyväskylä, Nanoscience Center, Department of Biological and Environmental Science, 40014 Jyväskylä, Finland and*
[b] *Institute of Biochemistry, Graz University of Technology, Petersgasse 12/II, 8010 Graz, Austria*

TABLE S1: The primers for the codon optimized sequences used for PaaC and PaaC +7 Y263F.

Forward:	5'-ATGCAGTTTCTGCGTAATATGGGTGTTGGTAGCAGC
Reverse:	5'-ACGCAGAAACTGCATGTGCATCGGGCTGG

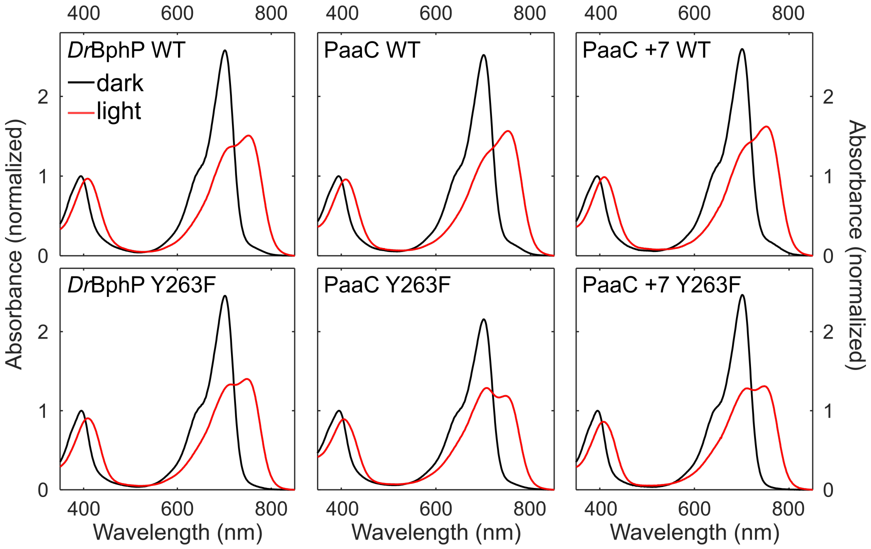

FIG. S1: Dark and light state UV-vis spectra of all the full-length *Dr*BphP, PaaC and PaaC +7 variants in this paper were measured to verify that all variants switch between the Pr and Pfr states. The spectra show that all the variants populate Pr and Pfr states in dark and red light, respectively. In Pr, the A_{700}/A_{280} ratio varies between the samples, but the spectral shape and maximum at 700 nm match the conventional Pr state spectrum.[1,4,5] The spectra are normalized at the maximum of the Soret band (394 nm) in the dark state. Dark spectra are acquired from samples kept in the dark or after far-red light (780 nm) illumination. Light samples are measured after saturated 661 nm LED illumination.

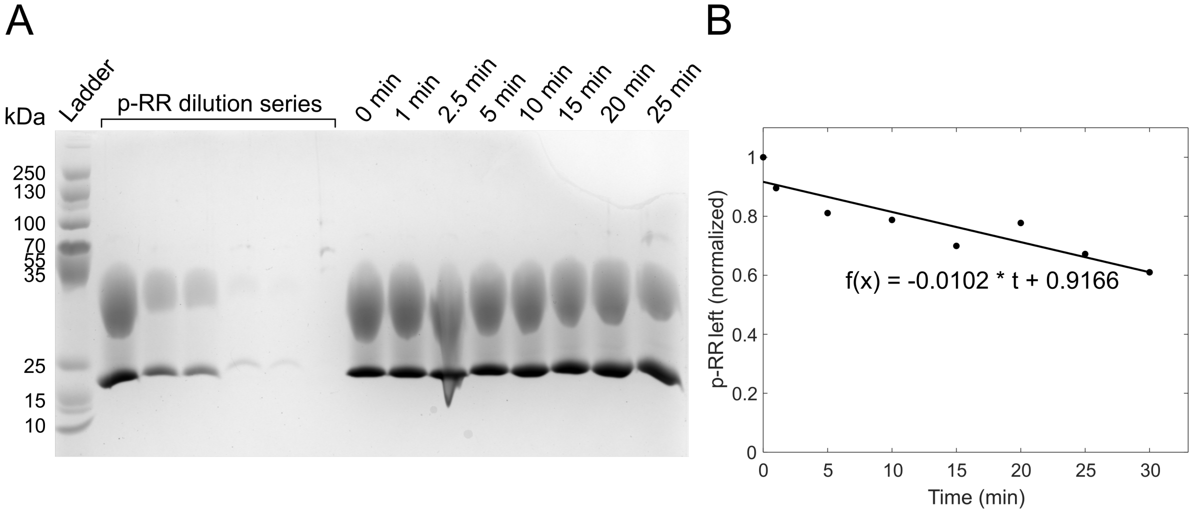

FIG. S2: A) 100% chemical phosphorylation of the response regulator (RR) population is not possible with our current methods. To determine the concentration of the phosphorylated RR (p-RR) a dilution series was performed. By determining the intensity of each band, 50.7% phosphorylation rate was revealed. Time series of p-RR (incubation time at +25°) follows the spontaneous breakage of p-RR. B) The intensities of p-RR bands in A were determined and normalized according to 0 min time point (dots). The single polynomial fit (solid line) was used as a correction line in determining the specific activity of *Dr*BphP (Fig. S4). The 2.5 min time point was not used and the 30 min time point is taken from a separate gel (Fig. S3 bottom left) with 0 min and 30 min time points.

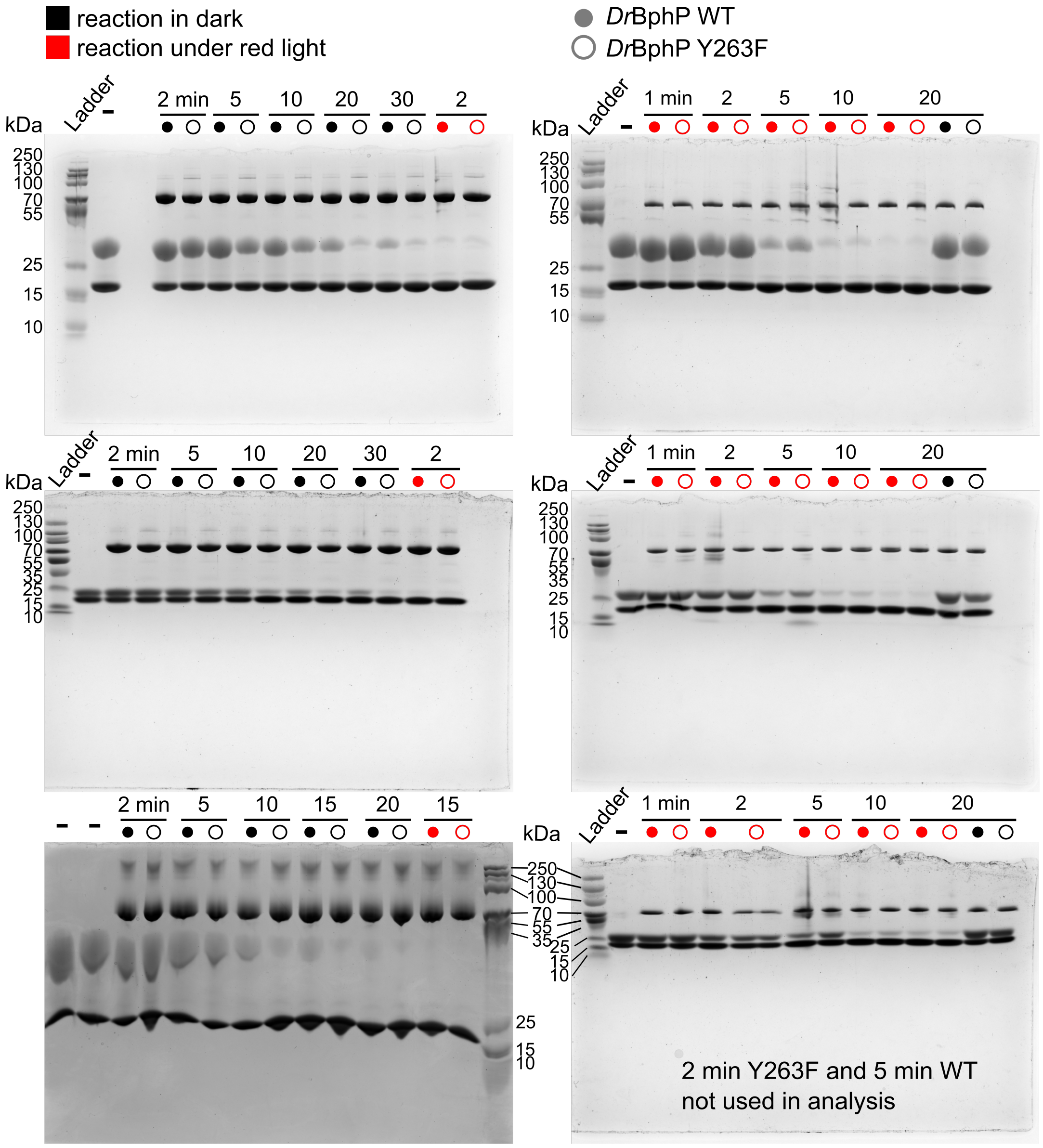

FIG. S3: Full images of PhosTag gels used in the analysis in Figures S4 and 4B. The molecular weight of *Dr*BphP response regulator (RR) is 84 kDa and 19 kDa, respectively. The phosphorylated RR migrates slightly slower than RR due to the PhosTag® in the gel, and is detected between the *Dr*BphP and RR bands.

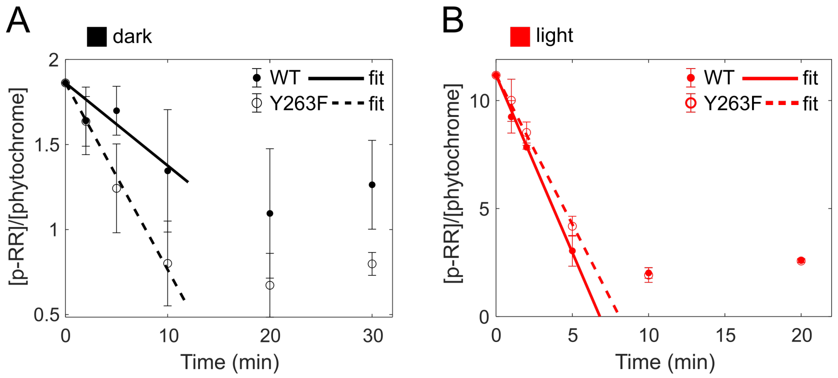

FIG. S4: The p-RR/DrBphP concentration ratio in each time point plotted against the time. The ratio is calculated based on the averaged band intensities in Fig. S3, and the standard deviation is presented as error bars. The initial p-RR concentration was calculated from Fig. S2A and the correction line (in Fig. S2B) due to spontaneous p-RR breakage in time was applied. The initial velocity V_0 is was determined from the first four time points after which the reaction starts to slow down considerably due to decreasing substrate concentration and competing RR/p-RR binding to the DrBphP[2]. The slope of the linear fit (lines) results as the specific activity of the construct either in dark or red light conditions, and are presented in Figure 4B.

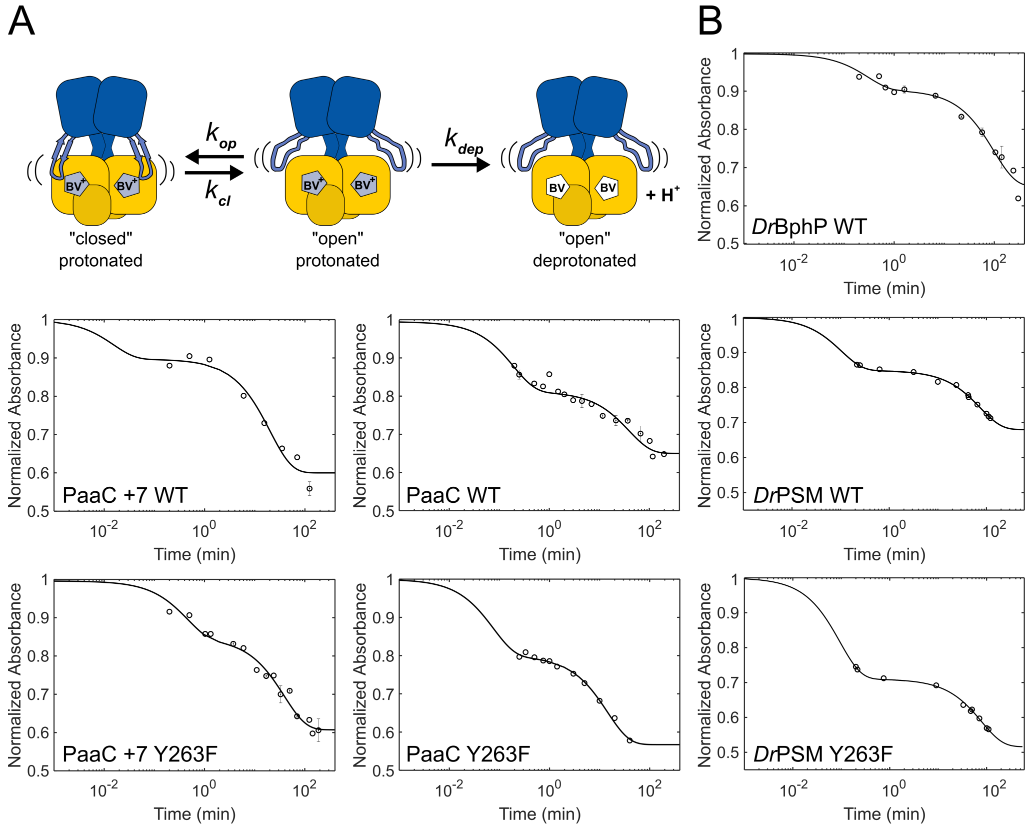

FIG. S5: A) In the DrBphP systems, the solvent access to the chromophore binding pocket is gated by the tongue[3]. When folded as β-sheet, it protects the BV from the solvent ("closed" conformation). Random coil or α-helical conformations allow the solvent to access the protein interior ("open" conformation). In most bacteriophytochromes, the BV in Pr state (*ZZZssa*) is mostly in the protonated state at pH 8. When the solvent pH is rapidly increased above the BV pKa, the BV deprotonates but only once the solvent reaches the BV. The rate of deprotonation depends on the the tongue dynamics and takes place in two phases. In the fast phase (with rate k_{dep}), all the phytochromes in "open" conformation deprotonate. The slow phase is limited by the tongue fluctuations between "closed" and "open" conformations with rates k_{op} and k_{cl}. B) The deprotonation is observed as decreasing of the 700 nm absorption and can thus be followed as a function of time with UV-vis spectroscopy (dots). The amplitude of the fast phase from the total amplitude of the deprotonation is the fraction of phytochromes in "open" conformation and thus reports on the stability of the tongue. We recorded the slow phase the same way as described in[3] to scale the fast phase in Fig. 2. Measured with manual mixing (dead time of 13 s), the amplitudes of the fast phase shown here are within the error margins of the slow experiment in comparison to our stopped-flow results.

* Electronic address: andreas.winkler@tugraz.at,janne.ihalainen@jyu.fi

[1] Auldridge, M.E., Satyshur, K.A., Anstrom, D.M., Forest, K.T., 2012. Structure-guided Engineering Enhances a Phytochrome-based Infrared Fluorescent Protein. J. Biol. Chem. 287, 7000–7009.

[2] Multamäki, E., Nanekar, R., Morozov, D., Lievonen, T., Golonka, D., Yuan Wahlgren, W., Stucki-Buchli, B., Rossi, J., Hytönen, V.P., Westenhoff, S., Ihalainen, J.A., Möglich, A., Takala, H., 2021. Comparative analysis of two paradigm bacteriophytochromes reveals opposite functionalities in two-component signaling. Nat. Commun 12. doi:\path{10.1038/s41467-021-24676-7}.

[3] Rumfeldt, J.A., Kurttila, M., Takala, H., Ihalainen, J.A., 2021. The hairpin extension controls solvent access to the chromophore binding pocket in a bacterial phytochrome: a UVvis absorption spectroscopy study. Photochem. Photobiol. Sci 20, 1173–1181. doi:\path{10.1007/s43630-021-00090-2}.

[4] Takala, H., Lehtivuori, H., Hammarén, H., Hytönen, V.P., Ihalainen, J.A., 2014. Connection between absorption properties and conformational changes in Deinococcus radiodurans phytochrome. Biochemistry 53, 7076–7085.

[5] Wagner, J.R., Zhang, J., von Stetten, D., Günther, M., Murgida, D.H., Mroginski, M.A., Walker, J.M., Forest, K.T., Hildebrandt, P., Vierstra, R.D., 2008. Mutational analysis of Deinococcus radiodurans bacteriophytochrome reveals key amino acids necessary for the photochromicity and proton exchange cycle of phytochromes. J. Biol. Chem. 283, 12212–12226.
